# Beta Test of a Christian Faith-Based Facebook Intervention for Smoking Cessation in Rural Communities (FaithCore): Development and Usability Study

**DOI:** 10.2196/58121

**Published:** 2024-08-26

**Authors:** Pravesh Sharma, Brianna Tranby, Celia Kamath, Tabetha A Brockman, Ned Lenhart, Brian Quade, Nate Abuan, Martin Halom, Jamie Staples, Colleen Young, LaPrincess Brewer, Christi Patten

**Affiliations:** 1 Department of Psychiatry and Psychology Mayo Clinic Health System Eau Claire, WI United States; 2 Department of Psychiatry and Psychology Mayo Clinic Rochester, MN United States; 3 Center for the Science of Health Care Delivery Mayo Clinic Rochester, MN United States; 4 Rural Health Research Core Center for Clinical and Translational Science Mayo Clinic Rochester, MN United States; 5 Living Water Church Cameron, WI United States; 6 Bethesda Lutheran Church Eau Claire, WI United States; 7 Valleybrook Church Eau Claire, WI United States; 8 St. John’s Lutheran Church (ELCA) Bloomer, WI United States; 9 Renew Church Eau Claire, WI United States; 10 Mayo Clinic Connect Health Education & Content Services Mayo Clinic Rochester, MN United States; 11 Department of Cardiovascular Medicine Mayo Clinic Rochester, MN United States

**Keywords:** social media, Facebook, rural, smoking, cessation, quitline, community-based participatory research, CBPR, FaithCore, mobile phone

## Abstract

**Background:**

Individuals living in rural communities experience substantial geographic and infrastructure barriers to attaining health equity in accessing tobacco use cessation treatment. Social media and other digital platforms offer promising avenues to improve access and overcome engagement challenges in tobacco cessation efforts. Research has also shown a positive correlation between faith-based involvement and a lower likelihood of smoking, which can be used to engage rural communities in these interventions.

**Objective:**

This study aimed to develop and beta test a social intervention prototype using a Facebook (Meta Platforms, Inc) group specifically designed for rural smokers seeking evidence-based smoking cessation resources.

**Methods:**

We designed a culturally aligned and faith-aligned Facebook group intervention, FaithCore, tailored to engage rural people who smoke in smoking cessation resources. Both intervention content and engagement strategies were guided by community-based participatory research principles. Given the intervention’s focus on end users, that is, rural people who smoked, we conducted a beta test to assess any technical or usability issues of this intervention before any future trials for large-scale implementation.

**Results:**

No critical beta test technical and usability issues were noted. Besides, the FaithCore intervention was helpful, easy to understand, and achieved its intended goals. Notably, 90% (9/10) of the participants reported that they tried quitting smoking, while 90% (9/10) reported using or seeking cessation resources discussed within the group.

**Conclusions:**

This study shows that social media platform with culturally aligned and faith-aligned content and engagement strategies delivered by trained moderators are promising for smoking cessation interventions in rural communities. Our future step is to conduct a large pilot trial to evaluate the intervention’s effectiveness on smoking cessation outcomes.

## Introduction

### Background

People living in rural communities experience significant disparities related to tobacco use cessation treatments due to geographic and infrastructure difficulties [1,2]. Rural areas have a higher prevalence of tobacco cigarette smoking compared to urban areas. This trend is observed in both men (29% vs 19%) and women (25% vs 13%) living in rural areas [3]. Owing to these inequalities, people living in rural communities experience a higher prevalence of chronic conditions, including cardiovascular disease and cancer [4,5]. In addition, rural populations have lower access to in-person (face-to-face) cessation services due to greater distance from clinics, which adds to the travel costs to access these services [6,7].

Social media and other digital platforms can potentially increase access and help overcome geographic and location-based barriers to engagement in tobacco cessation efforts, particularly in underserved populations such as rural communities [8]. Facebook (Meta Platforms, Inc) is the most popular social networking platform, with 68% of US adults using it. This is more than double the proportion of people on other social media sites, such as X (21%), Instagram (28%), Pinterest (26%), and LinkedIn (25%) [9]. Moreover, 75% of Facebook users engage with the platform daily, indicating high engagement [9]. Facebook is available 24/7 and has interactive tools that can engage users and foster peer support (eg, sharing personal experiences, offering hope, and increasing feelings of empowerment), which are crucial in substance use disorder treatment [10]. This can lead to greater intervention adoption and sustainability [11,12]. Therefore, developing an intervention that can be delivered through Facebook could encourage collaborative efforts to promote smoking cessation interventions and could resonate with rural populations.

Numerous studies observed the impact of spirituality and religious beliefs on positive health behaviors. Specifically, research has shown a positive correlation between this belief and a lower likelihood of cigarette smoking [13-15]. However, the definitions of religiosity and spirituality change across cultures and can even intersect; therefore, it has been challenging to define these terminologies, and there has not been any consensus on their definitions [16]. Although distinct concepts, religiosity and spirituality often share a core focus on the transcendent. Both involve believing in the supernatural, the sacred, or an “ultimate reality” beyond the physical world [16,17]. More specifically, religiosity refers to a codified and affirming relationship with institutionalized religions. Typically, religions have codified the set of doctrinal beliefs and prescriptive behaviors shared within a community of its adherents. By contrast, spirituality emphasizes the individual’s search for or attachment to the holy or ultimate reality. This may be outside of the domain of any religion; spiritual fulfillment within persons is achieved through practices such as meditation, associating with nature, and building a personal relationship with a higher power [18-21]. Simultaneously, “faith” is often described as a philosophical concept. Studies show that faith is a nonstatic concept and often means a belief pattern that provides meaning to them. Through faith, individuals try to gain an appreciation of their world and circumstances [22]. A religious scholar, Reza Aslan [23], defines and differentiates faith from religion by stating, “Faith is personal and mysterious and individualistic and inexpressible and indefinable. Religion is merely the language that you can use to express what is fundamentally inexpressible, to define what is undefinable” [24]. This study used a “faith-based” terminology to separate from religion and spirituality and align with the Centers for Disease Control and Prevention’s “faith-based initiative” for smoking cessation [25,26].

As a participatory approach and for the abovementioned reasons, collaborating with faith-based leaders can be highly effective in designing and testing interventions targeting health behaviors [27]. Community-based participatory research (CBPR) is an approach that involves collaboration with community stakeholders and partners at every step of the research. This approach allows community members to participate in all aspects of the research process and contribute their expertise with shared ownership and responsibility. Through this process, knowledge is exchanged, and action is integrated to improve the health and well-being of the community [28]. A review of clinical trials conducted in rural populations revealed that higher levels of engagement with communities toward CBPR approaches were associated with higher recruitment and participation rates [29]. Culturally relevant messaging for smoking cessation derived through CBPR enhances treatment acceptability [30,31], study recruitment, evidence-based smoking cessation treatment (EBCT) resource acceptance, and cessation rates [11,12,32]. This method is especially crucial for rural populations that are inaccessible and marginalized. This process empowers people to act and become agents of sustainable change in their communities, making a meaningful and lasting difference. This study operationalized intervention development through the informed strategic steps of CBPR to enhance sustainability and generate a more lasting impact within the rural community. The design process integrated the inclusion of key stakeholders such as faith leaders, academic researchers, and rural end users.

### Objectives

The FaithCore study aims to develop and beta test an innovative faith-based Facebook intervention prototype for rural individuals who smoked [2]. This paper describes the development and beta test of the Facebook intervention prototype. We also share critical lessons learned that could be applied by others interested in similar health behavior interventions.

## Methods

**Figure 1:**
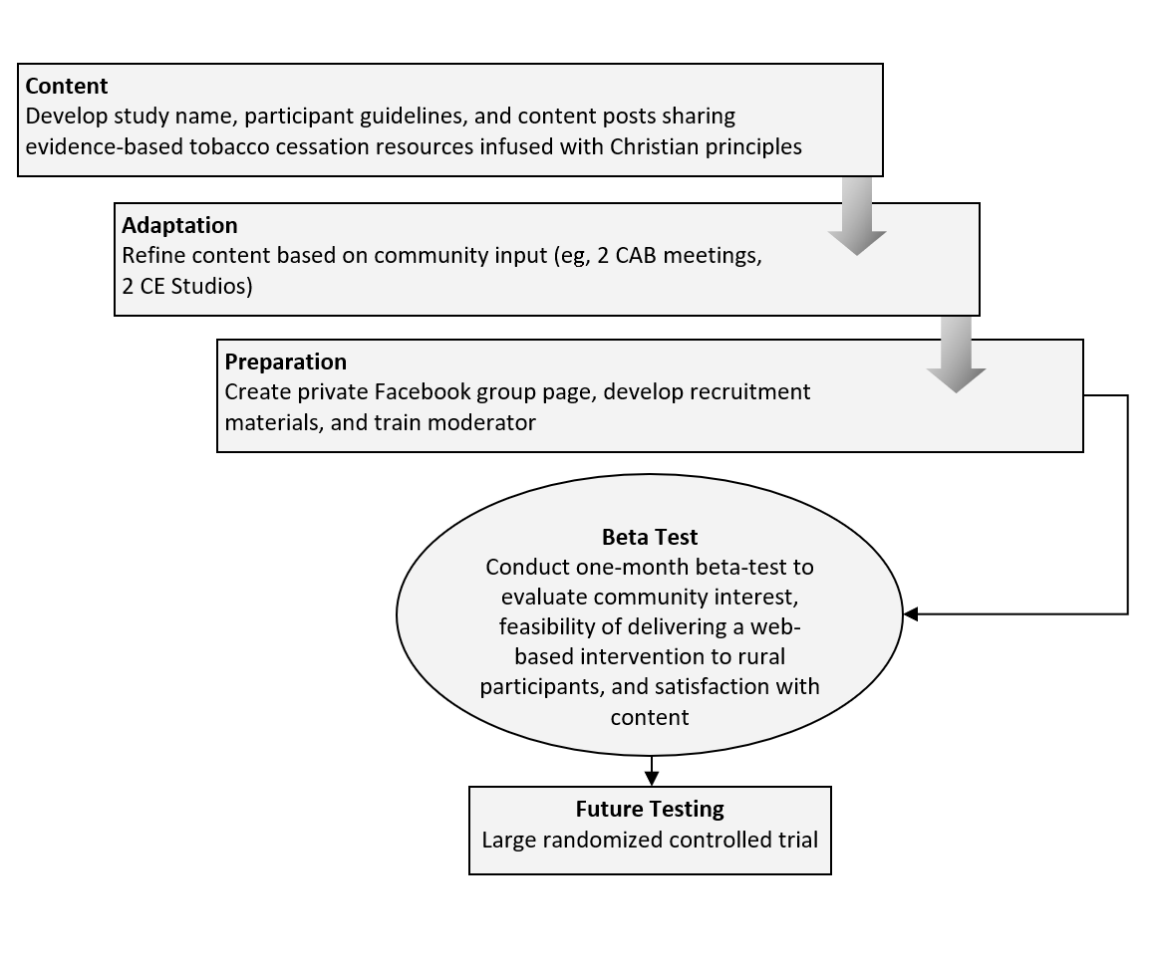
Overview of the development of the FaithCore intervention prototype and its beta test. CAB: community advisory board; CE: community engagement.

Figure 1 provides an overview of this study to develop and beta test the Facebook intervention prototype.

### Prototype Development

The formative work for the prototype development included (1) the creation of intervention materials, (2) community feedback on the intervention design and materials, and (3) intervention moderator training. The work of Pagoto et al [33] informed the development of the components of our prototype.

#### Community Engagement

We invited community members to join a community advisory board (CAB) and to participate in Community Engagement (CE) Studios. Before engaging with the community, the research team introduced CAB members with guidelines to establish and maintain a supportive, inclusive, and respectful community participation and sharing of opinions. These guidelines were built on our team’s previous work on this topic and that of Mayo Clinic’s Center for Health Equity and Community Engagement Research (CHCR). The CAB comprised 10 members (n=6, 60% women), including local pastors from Wisconsin, New Hampshire, and Tennessee, and people who smoked currently or in the past. The CHCR staff assisted in recruiting 5 CE Studio members (n=2, 40% women), consisting of community members representing potential end users (adult individuals who currently smoke) of the intervention. CAB members received an honorarium of US $150 per meeting. CE Studio members received honorariums from the CHCR.

#### First CAB Meeting

In May 2023, the study team and CAB held their first web-based meeting, which lasted for 90 minutes. The project lead, PS, provided a brief overview of the project and discussed respectful engagement guidelines to support CAB members when sharing feedback. Before meeting with CAB members, the research team that included rural health, substance use, and health disparities researchers; pastors from local churches; and rural patient advocates from the Mayo Clinic enterprise developed a preliminary content library. The posts were categorized by topic, such as connecting with others, practicing patience, prayer, and resisting temptation. Each topic had at least 3 posts that included an image or video, a relevant Bible verse, text, and a discussion post related to the topic. During the meeting, CAB members provided feedback on the preliminary content library on (1) Bible verses that were most suitable for the topic of each post, (2) images and videos that best reflected the content of each post and Bible verse, and (3) that the text of each post was culturally appropriate and not stigmatizing. The CAB feedback was incorporated, and posts were revised accordingly.

#### CE Studio

The study team, CHCR staff, and CE Studio community experts held a web-based 90-minute CE Studio. Overall, the CE Studio community experts appreciated the intervention content and agreed that if they were approached to participate in this study, they would be interested in joining a community faith-based Facebook group for smoking cessation. They also agreed that Facebook would be a good platform to receive this information, particularly because many rural people use it. However, some community experts mentioned that Facebook is not very appealing to a younger age group and that other social media platforms might be more effective in engaging young people who smoke. One participant noted, “For a younger age group, Facebook isn’t that appealing. I do not know anyone of my age (young adult) who uses Facebook anymore.” The CE Studio community experts emphasized the importance of incorporating Christian faith-based messages in smoking cessation–related posts. One of the participants stated, “This can help people of the Christian faith better understand their position when it comes to smoking and their faith. The content is motivating and good.” The CE Studio suggestions were discussed with the CAB members during the second meeting.

#### Second CAB Meeting

In August 2023, the study team held the second CAB meeting to review the feedback received from CE Studio community experts. In addition, we discussed a potential study logo designed by a local artist and beta test participant conduct guidelines for expected group behavior. The CAB members shared their thoughts on the feedback received and how to adapt the intervention materials. The changes made to the study materials before opening a beta test of the intervention are listed in Table 1. Table 2 provides an overview of refinements made to specific posts based on the CAB and CE Studio meetings.

**Table 1:** Summary of the second community advisory board (CAB) meeting feedback and resultant changes in the content library.

Study component and CAB feedback			Changes reflected in the content library
**Study name**			
	Original name: “Church-based social media intervention among rural people who smoke (CHURCH): Intervention prototype development.” The CAB members noted that not all Christians call their place of worship a church and not all individuals are members of a church. They liked “faith-based” and “community” to align with study goals.	New name: “FaithCore: A Christian Faith-Based Facebook Intervention for Smoking Cessation in Rural Communities”	
**Study logo**			
	Local artist designed the logo options. The CAB members requested design edits.	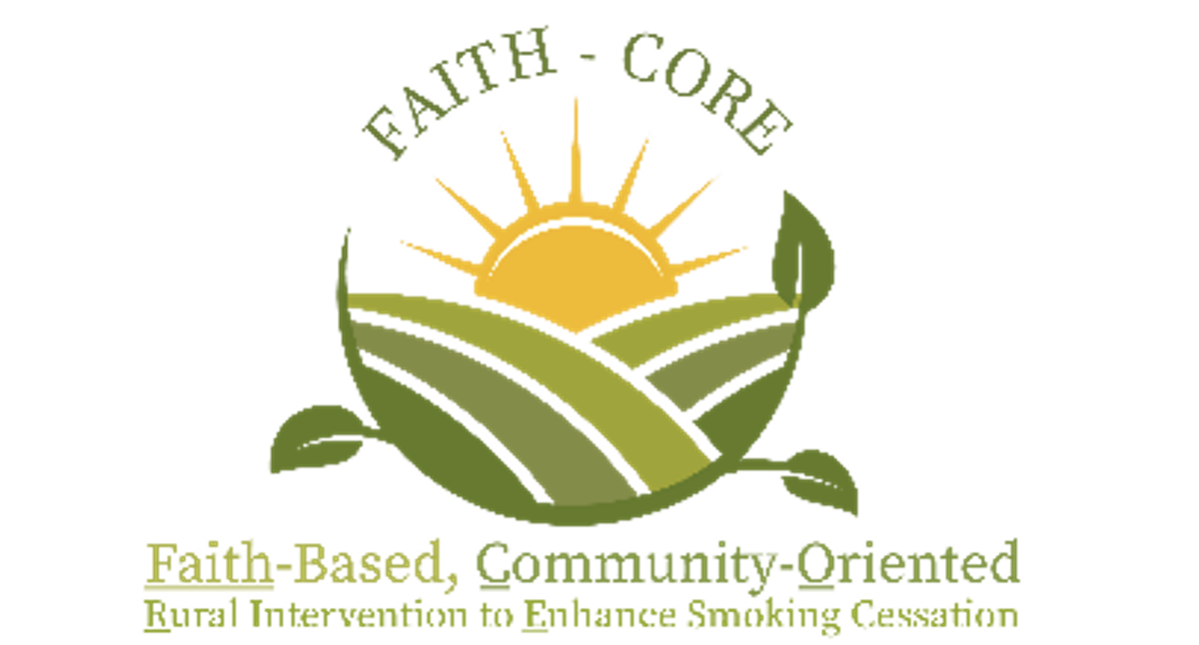 A new design was approved by the CAB members and used in study handouts and posts.	
**Facebook (Meta Platforms, Inc) intervention weekly topics**			
	Original weekly topics proposed: prayer, connection to others, grace, temptation, faith, and patience and forgiveness. The CAB members suggested that posts in this group should start with support. They recommended different topics for the weeks on NRTsa/medications (“grace”) and exercise (“faith”).	Weekly topics were narrowed to 4 weeks for the beta test: connection to others, patience, prayer, and temptation. NRT/medication posts were moved to the weekly topic of “patience.”	
**Facebook intervention posts**			
	The CAB members liked having simple posts and the inclusion of Bible verses and recommended adding a verse to each post/topic.	Weekly posts started with a separate “topic” post to make actual content posts simpler, and Bible verses were added to each post.	
	CAB members and pastors noted that mindfulness and meditation can be viewed negatively by some Christians.	Evidence-based research supports the efficacy of mindfulness in smoking cessation. A post from a well-known Christian organization was added, describing how Christians can use mindfulness in a biblical way.	
	The CAB members suggested photos of praying hands and people being mindful.	New stock photos or video link thumbnails were selected based on the recommendation.	
	The CAB members recommended adding encouragement for introverts to call the quitline.	The following text was added: “Some people are more introverted and don’t want to talk to a stranger. That’s ok! However, research shows that when you call a Quitline, you are 6 times more likely to quit than going cold turkey.”	
	The CAB members suggested incorporating confession.	The following verse was added to the quitline post: “Confess to one another and pray for each other so that you may be healed.” [James 5:16]	
**CE Studio feedback**			
	The CAB members did not agree with the CE Studio comment that “prayer was overused” and did not think stock photos would “make or break” the posts.	Some stock photos were replaced with other images or video link thumbnails.	
	The CAB members agreed with the recommendation to add a post about shame.	A post featuring a video from Dr Brene Brown was added to week 4 on reframing feelings of shame when struggling with cravings and relapse.	

^a^NRT: nicotine replacement therapy.

**Table 2:** Examples of revisions made to specific Facebook (Meta Platforms, Inc) posts before and after the community advisory board (CAB) feedback.

Topic	Before CAB feedback	After CAB feedback
Prayer	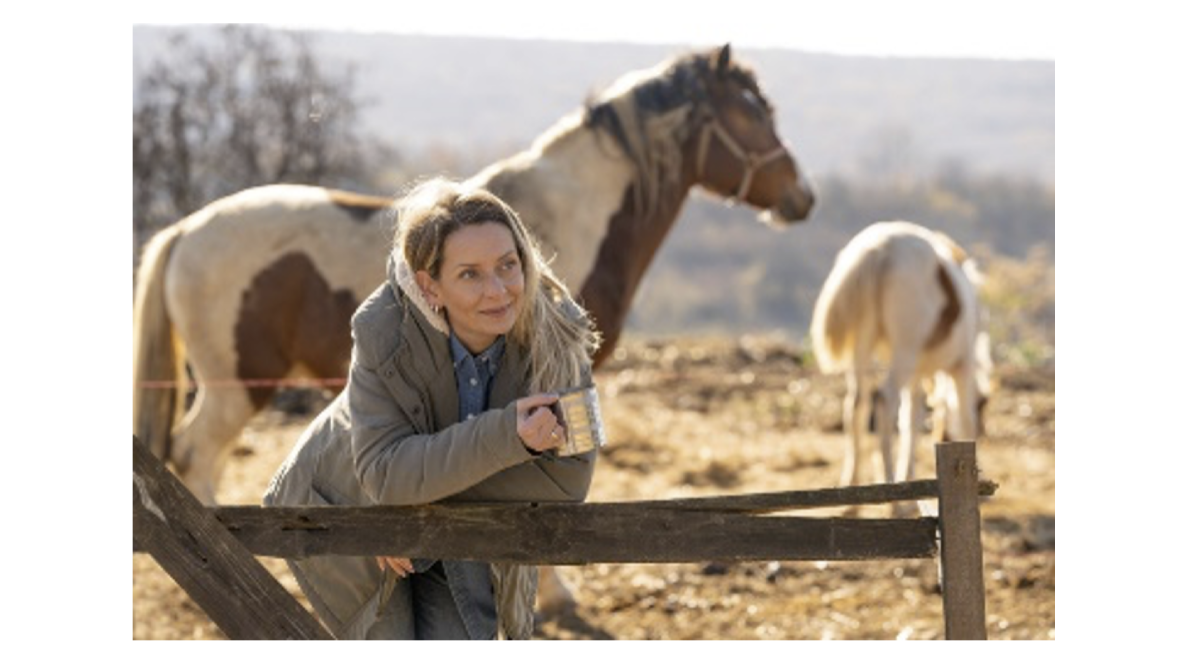 “Mindfulness is when we focus on the “right now” without judging ourselves or what we are sensing and feeling. We can practice mindfulness anytime, anywhere, while doing anything. Prayer itself is a mindful activity!” “Choose an activity you do every day – like washing dishes or brushing your teeth – and try doing it in a mindful, or prayerful, way.” “Video: Quit smoking through mindfulness” “Video: Mayo Clinic mindfulness living” “Discussion question: How do you think mindfulness can increase your awareness to stop and think before acting?”	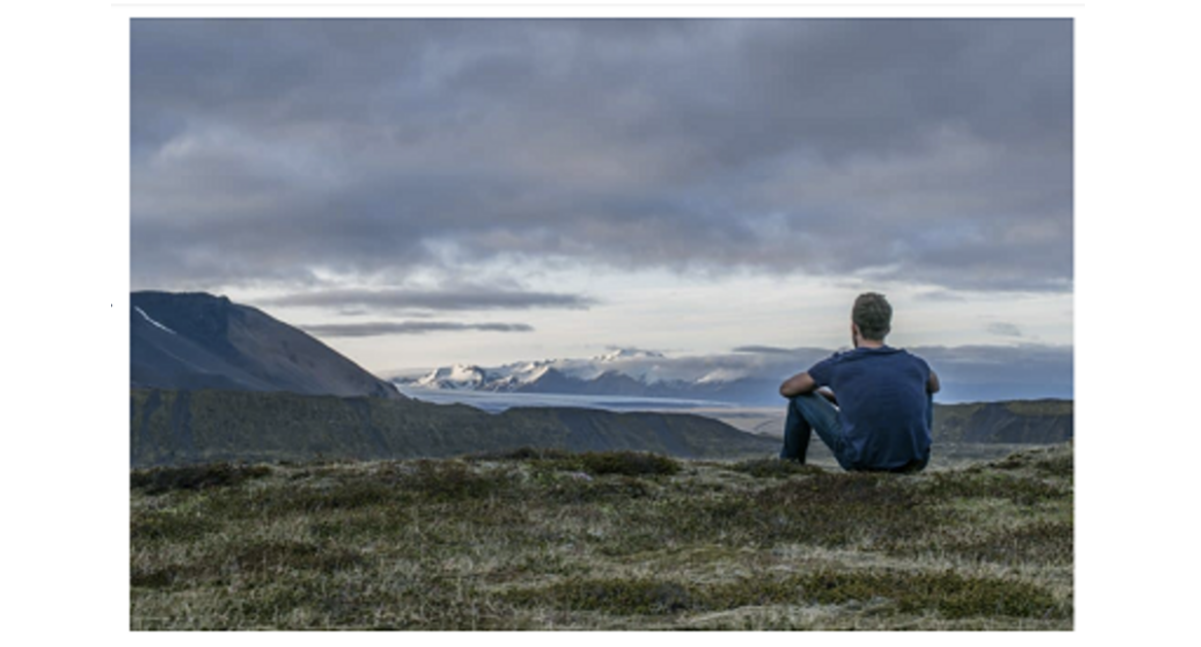 “Pray at all times in the Spirit.” Ephesians 6:18 “Mindfulness means focusing on the ‘right now’ without judging what we are sensing and feeling.” “We can practice mindfulness anytime, anywhere, while doing anything. Prayer itself can be a mindful activity!” “The article above discusses how Christians can use mindfulness from a biblical perspective: https://www.focusonthefamily.com/family-qa/mindfulness-a-christian-approach/” “Mindfulness is also effective in helping people quit smoking. The following webpage shares tips on using mindfulness to cope with cravings and stress: https://smokefree.gov/challenges-when-quitting/stress/practice-mindfulness” “Discussion question: How can you use mindfulness to be more aware, and pause before doing something out of habit or boredom?”
Patience	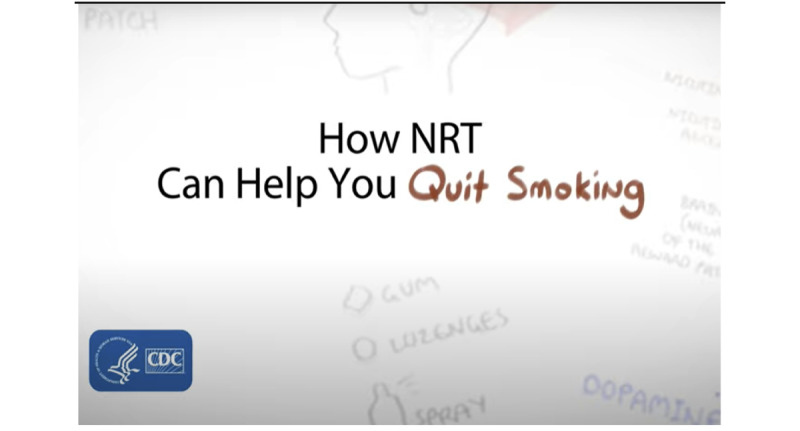 “You don’t have to quit cold turkey! In fact, more than 95% of smokers who quit cold turkey do not actually stay quit. There are many kinds of quit smoking medicines, and they each work in different ways. If you’ve tried one before and it didn’t work, we encourage you to check out this week’s posts and links to learn more about other types.” “There are 5 types of quit-smoking medications approved by the FDA [Food and Drug Administration]: Nicotine gum, patches, and lozenges (all available over the counter without a prescription) Bupropion (decreases cravings and other withdrawal symptoms; prescription needed) Varenicline (reduces the urge to smoke and reduces the pleasure from cigarettes if you do smoke; prescription needed)” “Video: How NRTs [nicotine replacement therapies] can help you quit” “Discussion question: How do you feel about relying on NRTs (patches, gum, medicines, etc.) to support you on your quitting journey?”	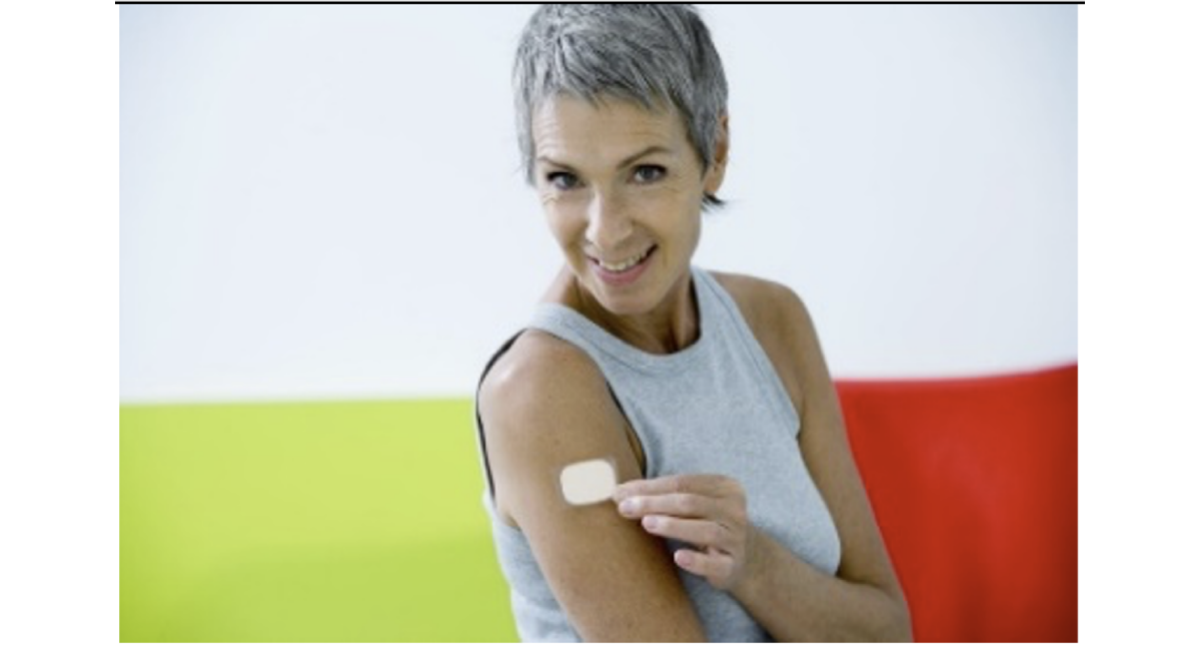 “Therefore I will boast all the more gladly about my weaknesses, so that Christ’s power may rest on me...For when I am weak, then I am strong.” (II Corinthians 12:9-10) “Our focus this week is on patience. Quitting smoking is hard. “Slipping,” having a cigarette, or even returning to smoking are a normal part of the quitting process. In fact, experts believe it takes most people at least 5-7 quit attempts before they finally stay quit. Go easy on yourself – you are not failing!” “Nicotine Replacement Therapies (NRT) can help you manage withdrawal while your body becomes healthy again. The following video explains how nicotine affects the brain and body, and how NRTs such as the patch, gum, and medications work: https://www.youtube.com/watch?v=g3Ar4v5K880” “This link has more information on NRTs and other quit methods to combine with medication: https://smokefree.gov/tools-tips/how-to-quit/using-nicotine-replacement-therapy” “Discussion question: In the past, have NRTs been successful in helping you manage withdrawal symptoms? If you’re new to NRTs, how can you plan ahead to use them?” “(Anonymous poll – Which NRTs have you tried [list]?”

### Preparation

#### Moderator Training

The study staff selected as a moderator for this study (author BT) completed training with author CY, Mayo Clinic social network director, in 2 parts, which spanned over 2 days. This comprehensive training provided a valuable introduction to the unique qualities of web-based groups and coached moderators on the principles of building a web-based community with purpose. The moderator was trained to foster conversations between members and how to engage members (beta test participants in this case) on the topic, intervention, and with one another. The strategies covered in the training were tailored to the program’s specific goals and tactics to achieve them. The training also included social media technical setup, welcoming members to the program, managing an active group, moderator self-care, and how to handle difficult situations. The training provided a predesigned reference guide to the moderator for future reference. CY remained available for the moderator to contact anytime for support.

#### Facebook Page Development

The study team responsible for conducting the beta test created a dedicated Facebook page. During the setup process, CY reviewed and approved various aspects of the page, including its design, study description, and welcome materials.

### Beta Test of the Intervention

A beta test is a small-scale field test comprising a preliminary feasibility study and a novel behavioral health intervention uptake. This field test allows for further refinement of the study protocol and the intervention itself before evaluating its efficacy. Beta testing’s primary objectives are to identify and address issues about intervention usability. By involving a group of target users (“end users”) as beta testers, researchers can gather valuable feedback regarding the quality and user experience of the intervention. This feedback is then used to make improvements before implementing a large-scale evaluation such as a randomized controlled trial. The purpose of this phase was to provide participants with 30 days of exposure to the FaithCore group and obtain their feedback to (1) ensure the system worked as intended, (2) identify technical issues, and (3) facilitate program refinements in preparation for the pilot testing.

### Study Recruitment and Methods

Participants were recruited using paid (sponsored) advertisements on Facebook and Instagram (Meta Platforms Inc), organic posts on the Mayo Clinic social media platform pages, and printed flyers sent to pastors on the CAB to post in church buildings. Interested participants were directed to a web-based eligibility screening survey hosted by Qualtrics (Qualtrics International Inc). Respondents screened but determined to be ineligible were given cessation resources and contact information for additional support. All participants provided written informed consent. At the end of the beta test, participants were asked to provide open-ended feedback on improving moderator posts and the Facebook group.

Study inclusion criteria were that participants (1) were aged ≥18 years; (2) lived in a rural area (based on rural-urban commuting area codes 4-10 [34] derived from zip codes) within the Mayo Clinic Health System catchment area; (3) had self-reported smoking at least 1 tobacco cigarette per day over the past 7 days; (4) were willing to make or consider attempting to quit smoking; (5) had reliable access to the internet on a computer, tablet, or smartphone (or were willing to use a loaner iPad for the duration of the study); (6) had or were willing to create a Facebook account; (7) were willing to complete a baseline survey before starting the intervention; and (8) were comfortable viewing study posts that contain Christian faith-based content, including Bible verses, and were willing to respect the confidentiality and faith perspectives of other group members. Participants were ineligible if they did not meet the abovementioned criteria. Our investigation recruited participants who were comfortable viewing and engaging with Christianity-based content, including Bible verses, but did not limit recruitment to any specific religious affiliation. Self-reporting as Christian was not part of the eligibility criteria.

### FaithCore Group Initiation

Building on our study team’s experience using web-based social media platforms to deliver behavioral interventions, we created a private Facebook group to share content posts with information on EBCT for smoking and connect group members for interpersonal support. Facebook allows us to host private groups that cannot be found in public searches and are only open to members by invitation from the group administrator. Posts in the group do not appear in the members’ main News Feed section or on their personal profile pages. To see and engage with group content, participants were instructed to access the group page at least 3 times per week during the 1-month intervention period.

Participants were informed during the consent that the group would start after 10 people had consented to the study [35]. They were asked to review participant guidelines and complete the baseline survey while waiting for others to join. One week before the intervention started, all the participants who consented were sent an invite and detailed instructions to join the private Facebook group. Upon entering the group, members were greeted with a welcome post and encouraged to introduce themselves to build connections with other members.

In December 2023, the intervention beta test was conducted over 4 weeks. The Facebook group was led by a moderator from the study team who had received training in facilitating web-based support groups. The moderator shared EBCT content posts 3 times per week for 12 posts on Mondays, Wednesdays, and Fridays. The weekly topics were connection to others, patience, prayer, and temptation. Because Christmas Day, a holy day in Christianity, fell on a Monday, the content post for that day was delayed until Tuesday, and a Christmas greeting post was shared on Monday.

Posts included an image at the top, a stock photo or a link thumbnail, a Bible verse, EBCT information related to that week’s topic, and additional links for more details. A discussion question was also posted as a comment to encourage conversations among group members. Participants could engage with posts by reacting (ie, like, love, care, laugh, surprised, sad, and angry), commenting and replying to others’ comments, and creating their own posts in the private group. The moderator also used engagement features such as anonymous polls, videos, and external links.

### Measures

Before joining the Facebook group, the participants who consented were asked to complete a baseline survey assessing demographics, including religious affiliation and tobacco cessation treatment in the past 30 days; the readiness to quit contemplation ladder [36]; an adapted Fagerström Test for nicotine dependence [37]; and a modified (for this study) brief version of the World Health Organization’s Quality of Life Measurement Instrument Spirituality, Religiousness, and Personal Beliefs facets [38].

At the end of the intervention, participants who had joined the Facebook group were asked to complete a survey assessing whether they had attempted to quit smoking during the program and any resources they used; the number of cigarettes in the past week; the 30-item Usefulness and Ease of Use Questionnaire [39], which was rated on a 7-point Likert scale; and open-ended questions on suggestions for improvement and overall satisfaction. Both the baseline and follow-up surveys were completed via REDCap, and participants received a US $25 cash card for completing each survey.

Beta test feasibility was measured by the number of consented participants who could join the Facebook group and by the number of members who actively engaged with the intervention (eg, view posts, react, comment, and create independent posts). At the end of the beta test, participants were also asked to provide open-ended feedback on improving moderator posts and the Facebook group.

### Data Analysis

Survey responses were collected on REDCap (Research Electronic Data Capture; Vanderbilt University) [40], which is a secure data management platform compliant with the Health Insurance Portability and Accountability Act. Descriptive engagement statistics were pulled from the insights summary available to group administrators, and additional details were compiled by the moderator (BT). We analyzed the responses to open-ended questions using content analysis [41].

### Ethical Considerations

The Mayo Clinic Institutional Review Board (23-000837) deemed the study exempt in February 2023, and the beta test phase was approved in October 2023. The CAB members received a US $150 honorarium, and CE Studio community experts received US $50. The participants received US $25 for completing the baseline assessment and US $25 for the postintervention assessment. For the beta test phase, study data were deidentified.

## Results

### Baseline

Recruitment was completed from November 14 to 20, 2023. A total of 31 interested participants were screened and 17 (55%) were eligible. The most common reason for ineligibility was not living in a rural area. Of the 17 participants, 12 (71%) provided written consent and were enrolled, and all 12 participants completed the baseline survey (11/12, 92% women; 11/12, 92% White; and 8/12, 67% employed part time or full time; 11 participants provided age with an average of 48.5 years; range 24-66 years; SD 13.1). Table 3 provides participants’ baseline characteristics.

**Table 3:** Baseline characteristics of the participants in the beta test. The participants were self-reported adult individuals who smoked and lived in rural areas (N=12).

Baseline characteristics			Values
Age (y; n=11); mean (range; SD)^a^			48.55 (24-66; 13.1)
Gender (women), n (%)			11 (92)
**Ethnicity or race, n (%)**			
	African American	1 (8)	
	White	11 (92)	
**Marital status, n (%)**			
	Single, divorced, or separated	7 (58)	
	Married or long-term relationship	5 (42)	
**Employment status, n (%)**			
	Part time or full time	8 (67)	
	Homemaker	2 (17)	
	Retired	1 (8)	
	Other^b^	1 (8)	
**Education, n (%)**			
	High school or GED^c^	1 (8)	
	Some college	1 (8)	
	Associate’s degree	5 (42)	
	Bachelor’s degree	4 (33)	
	Graduate degree	1 (8)	
**Currently a member of an organized religious group, n (%)**			
	Yes	7 (58)	
	No	5 (42)	
**Frequency of attending organized religious events, n (%)**			
	Once a week or more	2 (17)	
	About 2-3 times a month	1 (8)	
	About once a month	1 (8)	
	A few times a year	2 (17)	
	Almost never	1 (8)	
**Faith tradition (options also included Buddhist, Muslim, Jewish, and other), n (%)**			
	Catholic	3 (25)	
	Evangelical	2 (17)	
	Lutheran or Protestant	7 (58)	
Used cessation treatment or medication to quit smoking in the last 30 days, n (%)			2 (17)^d^
Quit attempt in the last 30 days, n (%)			9 (58)
**Intended time frame to quit smoking, n (%)**			
	Within the next month	7 (58)	
	Within the next 6 months	4 (33)	
	Someday, but not in the next 6 months	—^e^	
	Not sure	1 (8)	
Contemplation ladder score (1=not ready and 10=ready now), mean (range)			7 (5-10)
Confidence in the ability to quit (1=not at all and 10=completely), mean (range)			6 (3-8)
Minutes to first cigarette of the day within 5 minutes, n (%)			4 (33)
**How many cigarettes per day do you smoke? n (%)**			
	≤10	5 (42)	
	11 to 20	5 (42)	
	21 to 30	3 (25)	
	≥31	—	

^a^Data were missing for 1 participant.

^b^Works for 3 to 4 hours per week.

^c^GED: General Education Development.

^d^One participant had used nicotine replacement therapy, and 1 participant had used non-nicotine medication.

^e^No data.

### Spirituality and Religion

All participants responded that spirituality has at least “a little” importance in their lives. Each participant noted the importance of religion in their overall identity. No participant responded that spirituality and religion are not important to them.

### Facebook Group Activity

Of the 12 participants, 2 (17%) could not join the Facebook group and reported technical difficulties with the internet or their Facebook app and were withdrawn from the study. Of the 12 participants, 10 (83%) joined the private Facebook group and participated actively throughout the beta test. All participants viewed the posts, made comments, and reacted to posts at least 3 times. A total of 8 (80%) of the 10 participants posted comments on multiple posts and engaged with each other to share personal experiences using nicotine replacement therapies, non-nicotine medications, and different coping strategies as well as to celebrate their success in cutting back or quitting using the new information shared through the intervention. As anticipated, engagement slowed somewhat over the week between Christmas and New Year, but all participants continued to enter the group and view posts. No participant reported problems or technical difficulties accessing or participating in the group.

### End-of-Intervention Survey

At the end of the beta test, 90% (9/10) of participants self-reported that they had tried to quit smoking tobacco cigarettes since joining the FaithCore group. Furthermore, 90% (9/10) of participants reported using or seeking EBCT resources. Of those who sought treatment, 60% (6/10) reported using nicotine replacement therapies, 20% (2/10) called a quitline, 20% (2/10) sent SMS text messages to a quitline, 40% (4/10) used counseling services, and 40% (4/10) used group support services.

Responses on the Usefulness and Ease of Use Questionnaire showed that participants found the Facebook group easy to use and were satisfied with it. On the 1 to 7 Likert scale, mean scores in the following categories were usefulness 5.52 (SD 1.18; range 2-7), ease of use 6.01 (SD 1.17; range 2-7), ease of learning 6.47 (SD 0.89; range 4-7), and satisfaction 5.86 (SD 1.16; range 3-7).

### Participant Feedback on Group

Participants provided open-ended feedback on improving moderator posts and the Facebook group (Textbox 1). Four areas of response emerged: (1) increasing the number of participants (>10 before the group starts), (2) encouraging participants to share additional information about themselves, (3) making posts more interactive, and (4) encouraging group members to post more frequently. Moderator expressed no concerns with social media engagement and moderating the group.

**Quotes from FaithCore beta test participants at the end-of-study survey.**

**Suggestions for improving the post content (eg, text, images, verses, and links)**

- “More posts in general, more scripture more pictures”
- “Run program longer with a larger group of people”
- “I think the group members could’ve been more active”

**Suggestions for improving discussion questions or interactions with group members**

- “Maybe a group chat where we got to know each other better”
- “Use polls more”
- “Make the questions shorter and more interactive”

**Suggestions for overall improvement**

- “More posts in general, then I would see it more and interact more”
- “Great idea! More people would benefit from this. More conversations daily would be fabulous”
- “More members”
- “More specific activities to try”

**Positive aspects of the program**

- “Positive and friendly”
- “Good content, nice to talk to others”
- “I liked that it was always there, so easy to use”
- “Enjoyed the support”
- “I found extra techniques to add to my quitting arsenal”
- “They were kind. Everyone was willing to help and listen to each other”
- “It was a wholesome community and we all struck to the plot of the group, everyone encouraged each other”
- “They inspired me to continue”
- “Interesting articles and resources”

**Negative aspects of the program**

- “I think it was a bit boring. Probably because we had few members”
- “Didn’t love having my name and business out there”
- “Would have liked more interaction; seemed to focus on meds”

## Discussion

### Principal Findings

This paper describes a CBPR process to create and beta test a social media intervention prototype aimed at helping people quit smoking. The prototype was designed to provide evidence-based resources, peer support, and culturally tailored content. The paper discusses various strategies that were used to engage users, train the moderator, and create content, aiming to develop a prototype for a pilot testing trial.

The intervention prototype was developed using the CBPR approach, and it met all the set goals and timelines. The project was completed smoothly, with no concerns raised by the CAB and CE Studio members. Regular updates to community members and stakeholders on the study’s progress played a vital role in the project’s success. Similar to our earlier work with social media platforms for addiction treatment [12], the FaithCore beta test was feasible. In the beta test phase, we used validated social media usability measures, and participants scored high in every category (ie, usefulness, ease of use, ease of learning, and satisfaction), with an average mean score of >5. This indicates that the intervention was useful, easy to learn, and satisfies its intended purpose.

In addition, there was a potential effectiveness signal, with 90% (9/10) of the participants attempting to quit smoking tobacco cigarettes and 90% (9/10) of them reporting using or seeking the cessation resources discussed in the group to help them quit smoking. The qualitative feedback during the beta test will facilitate specific improvements to the prototype and intervention before conducting larger pilot trials. During the beta test study, 2 (17%) of the 12 participants dropped out due to technical problems. Because digital disparities are prevalent among rural residents, strategies should be planned to assist participants as needed for digital access and digital literacy challenges [42]. For the future pilot phase, we plan to have a digital navigator on board for unforeseen digital connectivity problems that participants may experience.

The primary feedback from the beta test participants was to increase interaction and engagement among the group participants. Participant engagement in the group is critical to exchanging experiences and supporting one another actively. As we prepare for the pilot phase, we will discuss strategies to augment participant engagement with social media experts and the moderator in our study. One way to increase engagement could be incentivizing the participants to comment on the post. For example, Lyu et al [43], in a social media study, incentivized participants to comment on Facebook posts to increase group engagement. In this study, the constructs of spirituality and religiosity were not the primary focus. It is postulated that these constructs evolve with personal life experiences, which often influence their future course. For example, individuals may maintain their Christian affiliation while altering their level of participation in religious activities. Similarly, bereavement, illness, or major life transitions can lead to a re-evaluation of spiritual beliefs and practices [18-21]. Furthermore, there is often a delicate balancing act between religious beliefs and spirituality, which may positively impact one’s health behaviors, such as engaging in smoking cessation. Our future pilot study will periodically administer validated instruments to assess spirituality and religiosity. This will be used to examine the modifying influence of these constructs on our main results regarding smoking reduction behaviors and cessation outcomes.

### Limitations and Strengths

Given that the beta test participants were restricted to the Midwest population, the acceptability of this intervention may limit its generalizability to other rural populations. However, this phase was focused on prototype development and testing the overall function of the prototype. Future pilot study steps will pave the way toward larger and more generalizable evaluations. For the beta test, we recruited individuals with access to the internet and smart devices to access Facebook, or were willing to use a loaner device with internet access, which may lead to selection bias. For future larger testing, we will consider providing a loaner digital device to participants with no digital access to eliminate this selection bias [42].

Our project has many strengths. We adopted a CBPR approach to develop our prototype, and we proactively engaged health communication and social media experts to train the moderator to achieve high-level engagement during beta testing. Throughout each step, we gathered multiple levels of feedback from the community and end users to improve the content library and conduct the project. While most of the feedback has already been incorporated, the beta test feedback will be used to further improve the process and content library before future pilot studies. Our community stakeholders expressed interest in being trained as moderators and maintaining the proposed Facebook group, ensuring self-sustainability after the study.

### Conclusions

In conclusion, this study suggests that social media group interventions cocreated through a CBPR approach, incorporating culturally aligned and faith-aligned content and engagement strategies, hold promise for promoting positive outcomes in rural populations when facilitated by trained moderators. The intervention prototype met all the set goals and timelines. During the beta test phase, we did not identify any critical technology-related or user interface–related issues. However, better digital support is needed for future trials targeting rural populations to prevent participant attrition rates. Further research and a large pilot trial are being planned to evaluate the effectiveness of this intervention prototype.

## Abbreviations

CAB: community advisory board
CBPR: community-based participatory research
CE: community engagement
CHCR: Center for Health Equity and Community Engagement Research
EBCT: evidence-based smoking cessation treatment
